# In Situ Synthesis of Copper Nanoparticles on Dielectric Barrier Discharge Plasma-Treated Polyester Fabrics at Different Reaction pHs

**DOI:** 10.1021/acsapm.2c00375

**Published:** 2022-04-15

**Authors:** Behnaz Mehravani, Ana Isabel Ribeiro, Uros Cvelbar, Jorge Padrão, Andrea Zille

**Affiliations:** † 2C2T—Centre for Textile Science and Technology, Department of Textile Engineering, University of Minho, Campus de Azurém, Guimarães 4800-058, Portugal; ‡ Department of Gaseous Electronics (F6), Jožef Stefan Institute, Ljubljana SI-1000, Slovenia; § Faculty of Mathematics and Physics, University of Ljubljana, Ljubljana SI-1000, Slovenia

**Keywords:** copper nanoparticles, multifunctional textiles, antibacterial textiles, coloration of polyester, UV protection, DBD plasma treatment

## Abstract

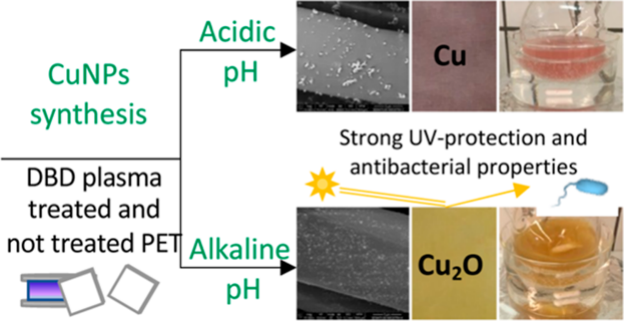

Polyester (PET) fabrics are widely applied in functional textiles due to their outstanding properties such as high strength, dimensional stability, high melting point, low cost, recyclability, and flexibility. Nevertheless, the lack of polar groups in the PET structure makes its coloration and functionalization difficult. The present work reports the one-step in situ synthesis of copper nanoparticles (CuNPs) onto the PET fabric employing sodium hypophosphate and ascorbic acid as reducing and stabilizing agents, at acidic (pH 2) and alkaline pH (pH 11). This synthesis (i) used safer reagents when compared with traditional chemicals for CuNP production, (ii) was performed at a moderate temperature (85 °C), and (iii) used no protective inert gas. The dielectric barrier discharge (DBD) plasma was used as an environmentally friendly method for the surface functionalization of PET to enhance the adhesion of CuNPs. The size of the CuNPs in an alkaline reaction (76–156 nm for not treated and 93.4–123 nm for DBD plasma-treated samples) was found to be smaller than their size in acidic media (118–310 nm for not treated and 249–500 nm for DBD plasma-treated samples), where the DBD plasma treatment promoted some agglomeration. In acidic medium, metallic copper was obtained, and a reddish color became noticeable in the textile. In alkaline medium, copper(I) oxide (Cu_2_O) was detected, and the PET samples exhibited a yellow color. The PET samples with CuNPs presented improved ultraviolet protection factor values. Finally, a minimal concentration of copper salt was studied to obtain the optimized antibacterial effect against *Staphylococcus aureus* and *Escherichia coli*. The functionalized samples showed strong antibacterial efficacy using low-concentration solutions in the in situ synthesis (2.0 mM of copper salt) and even after five washing cycles. The DBD plasma treatment improved the antibacterial action of the samples prepared in the alkaline medium.

## 1 Introduction

The functional textile sector has seen significant growth during the last few years due to the need for high-performance materials.^1^ The expectations for green, high-value, durable, comfortable, and functional products have created several opportunities to replace hazardous finishing chemicals and introduce novel functionalities, especially in medical and protective textile sectors.^2−4^ In this field, nanotechnology has earned great attention. It can impart coloration, stain repellence, wrinklefreeness, electrical conductivity, UV protection, static elimination, flame retardance, and antimicrobial action to fibers, without compromising their natural properties such as flexibility.^5−8^

Copper nanoparticles (CuNPs) have been used for a wide range of applications due to their unique optical, catalytic, antimicrobial, electrical, mechanical, and thermal properties.^9^ CuNPs have some advantages when compared with other metal nanoparticles (MNPs) due to their superior biocompatibility. Generally, an excessive release of metal ions in the human body can be harmful due to uncontrolled bioaccumulation. In the case of copper, the ions are transported by adenosine triphosphatase, which plays an essential role in copper elimination and increase the CuNPs’ biocompatibility. Moreover, copper has high natural abundance and is of low cost.^10,11^

Three methods have been commonly reported for MNP preparation: chemical, physical, and biological approaches.^12^ Chemical reduction is one of the most prevalent methods for CuNP synthesis due to the simplicity of controlling the nanoparticles’ shape and size.^13^ This method reduces the ionic salt with an appropriate reducing agent and commonly uses a capping agent to form the nanoparticles and inhibit agglomeration. However, most of the chemicals in the form of solvents, reducing agents, and stabilizing agents are expensive and harmful to human health and the environment. The most common reducing agents used in the chemical synthesis of CuNPs are sodium borohydride, hydrazine, isopropyl alcohol, and formaldehyde.^14,15^ The MNP synthesis using green processes is an emerging field and is a promising tool to overcome the disadvantages of traditional chemical reduction methods. Based on the values of green chemistry, there are some acceptable methods for synthesizing MNPs, which may include bacteria, fungi, yeasts, plant extracts, or non-toxic and eco-friendly chemicals as reducing agents. These syntheses must also be performed at atmospheric pressure and low temperatures.^16−18^ Sodium hypophosphite (SHP) has been described as a suitable alternative reagent in chemical synthesis because it presents low cost, relative safety, and allows the synthesis in the aqueous medium.^19−21^ In addition, ascorbic acid (AA), a biocompatible substance, may be added to act as a reducing agent and also as a capping agent, preventing the oxidation of CuNPs.^22,23^

The properties of polyester (PET) fabric, such as its high strength, dimensional and thermal stability, and excellent chemical properties, make it one of the preferred materials for high-performance textiles for medical and protective applications. However, the lack of polar groups on its backbone makes it highly hydrophobic, resulting in poor wettability and adhesion, making its coloration, functionalization, as well as proliferation of pathogens difficult.^24−26^ To improve the adhesion of MNPs, the PET has been functionalized with functional groups, namely, alcohol, carboxylic acid, and amine groups.^27^ Several researchers have reported the usage of surface modification techniques such as photo-induced irradiation, electron beam irradiation, enzymatic modification, alkaline hydrolysis, aminolysis, alcoholysis, and plasma treatments.^27,28^

Plasma treatment has been stated as an environmentally friendly and cost-effective technique to modify materials.^29^ This method involves the generation of reactive free radicals, photons, atoms, and ions, which change the surface and facilitate the incorporation of functional groups such as hydroxyls, carboxyls, amines, and amides. As a result, the adhesion of MNPs may be enhanced and may occur as hydrogen bonds, van der Waals forces, or dipolar interactions.^25^ In addition, microroughness of the surface can enhance the adhesion between fibers and coatings.^30^ Plasma-discharging parameters such as the type of gas, treatment time, and input power should be appropriately selected. Besides eco-friendliness, plasma treatments do not change the material’s bulk properties and only influence the outer layer of the substrate.^31^ When plasma is operated in air, without expensive carrier gases, this technique is very cost-effective. Oxidation reactions during plasma treatment in air generate polar functional groups that may improve the hydrophilicity of the material, creating highly reactive species on its surface. Additionally, reactive oxygen species (ROS) and reactive nitrogen species produced during the plasma treatment in air can also promote some antimicrobial action.^32,33^

Few reports were found in the literature considering PET fabrics and the in situ synthesis of CuNPs. In these studies, the authors commonly report the formation of CuNPs by chemical methods, where the size and shape of CuNPs strongly depend on the reducing agent used. The PET coloration is mediated by the shape, size, and oxidation state of the CuNPs. Moreover, good antibacterial properties can be found.^8,34−36^

In this study, untreated and dielectric barrier discharge (DBD) plasma-treated PET fabrics were functionalized with in situ synthesized CuNPs using three different concentrations. CuNPs were produced in two ways, that is, in acidic and alkaline media at moderate temperature. In acidic medium, AA and SHP were combined to prepare CuNPs. In an alkaline reaction, sodium hydroxide was additionally added to the synthesis. To the best of our knowledge, multifunctionalization of the PET fabric using DBD plasma treatment and different pH conditions for the in situ synthesis of CuNPs using both SHP and AA has not been reported. The red (acid pH) and yellow (alkaline pH) coloration of the fabrics was observed using the highest concentration tested (10.0 mM of copper salt). Moreover, UV protection and high antibacterial activities against *Staphylococcus aureus* and *Escherichia coli* were obtained even using a low concentration of CuNPs [starting from a five times diluted (5TD) solution of the copper salt and 2.0 mM of copper salt] and after at least five washing (5W) cycles. The samples’ characterization was performed by scanning electron microscopy (SEM) analysis, ultraviolet–visible (UV–vis) spectroscopy [measuring the color coordinates and reflectance (%)], thermogravimetric analysis (TGA), X-ray photoelectron spectroscopy (XPS), and X-ray diffraction (XRD). The developed PET fabrics are promising candidates for antibacterial wound dressing, medical textiles, or hygienic products to prevent cross-contamination and nosocomial infections.

## 2 Results and Discussion

### 2.1 PET Fabric Functionalization

**Figure 1:**
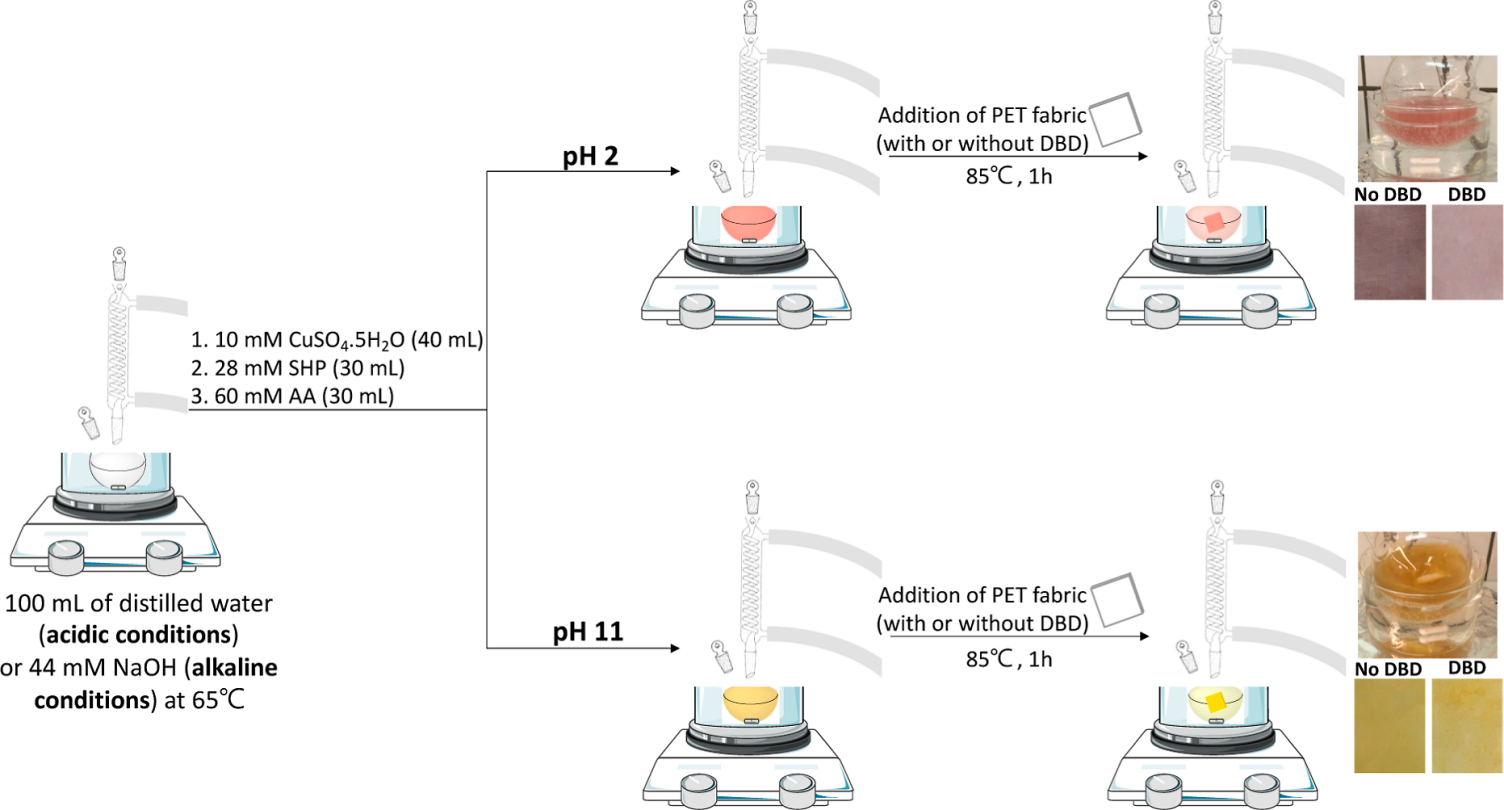
In situ synthesis of CuNPs under acidic (pH 2) or alkaline (pH 11) conditions onto DBD plasma-treated (DBD) and not treated PET fabrics (No DBD).

PET fabrics were functionalized with in situ prepared CuNPs in acidic and alkaline media without inert gas protection. In the acidic reaction, at pH 2.4, the CuNPs were formed by chemical reduction in water after mixing the copper sulfate (10 mM), AA (60 mM), and SHP (28 mM) (Figure 1—pH 2). The PET sample was immediately added to the flask when a light red color was noticed corresponding to the early first stage of CuNP nucleation, which was maintained during the entire growth stage of CuNP formation. The temperature was adjusted to 85 °C and kept for 1 h to promote a higher diffusion of the ions and/or smaller CuNPs into the fabric fibers. SHP and AA were both used as reducing agents. An excess of AA was used to reduce the copper salt and prevent further oxidation.^37^ Although both AA and SHP are used, since AA is a weaker reducer agent than SHP for the formation of CuNPs, the reduction reaction must be promoted mainly by SHP. However, AA has already been shown to bind to the surface of CuNPs, diffusing the species during the growth phase, allowing greater control over their subsequent oxidation and also promoting the spherical shape of the nanoparticles.^38^

The combination of a weak acid with a strong base has been reported as an excellent choice to control the growth rate and structural transformation of CuNPs.^39^ Thus, the addition of NaOH into the reaction was tested, promoting the alkaline reaction (pH 11). In this case, the emergence of a yellow-brownish color indicated the beginning of the reduction reaction (Figure 1—pH 11).

PET samples with and without DBD plasma treatment were used in acidic and alkaline reactions (pH 2-DBD/pH 11-DBD and pH 2-No DBD/pH 11-No DBD, respectively). The reactions in acidic and alkaline media were performed using three different concentrations of copper salt (10, 2, and 1 mM) and the corresponding reducing and stabilizing agents (Table S1 in the Supporting Information).

The synthesis using only AA as a reducing agent was attempted, but the CuNP dispersion oxidized in a few minutes, which was observed by a color change. The addition of SHP exhibited a critical prevention of the CuNP oxidation during the synthesis. Combining the reducing properties of SHP and AA, the oxidation was avoided and the velocity of the reduction increased. A black (acidic pH) or green (alkaline pH) color appeared if the addition of SHP was not immediate. Moreover, during the alkaline reaction, the fabric must be quickly added in an early stage of the reaction to prevent the emergence of the dark green color, attributed to superior oxidation states of copper. The color reverted to light yellow when the PET samples were added. When the fabric was not added, the NaOH reacted with AA and SHP, decreasing their reducing activity, thus a darker color developed. This color is commonly attributed to copper(II) oxide.^40^ Thus, the extra stabilization of copper ions in the presence of PET may be due to the alkaline hydrolysis of PET and corresponding NaOH consumption. Alkaline hydrolysis is one of the most common finishing methods for PET fabrics to enhance their wettability, dyeability, and silk-like texture. Under this condition, the hydroxyl ions from NaOH attack the electron-deficient carbonyl carbons of PET, followed by chain scission and creation of hydroxyl and carboxylate end groups. The reaction of PET with an alkaline agent promotes the formation of nano-sized rugosity and extra functional groups.^41^ In this case, the pH of the reaction also confirmed the functionalization (the pH decreased from 11 to 7.2). The produced hydroxyl groups of PET can act as a stabilizing agent by coordinating with the copper ions.^42^ Other works also describe the cationic ions’ essential role in neutralizing the negative surface charge of PET in the alkaline treatment, attracting more hydroxyl groups and leading to enhanced alkaline hydrolysis.^43^ Therefore, the alkaline hydrolysis and the use of copper salts can complement each other: on the one hand, the copper ions can neutralize the negative charge of the PET surface, attracting more hydroxyl groups to the PET surface (increasing the effect of the alkaline hydrolysis); on the other hand, the superior number of hydroxyl groups on the PET surface may attract more copper ions (leading to a superior stabilization of copper onto the PET surface).

### 2.2 Characterization of PET Samples

#### 2.2.1 SEM Analysis

**Figure 2:**
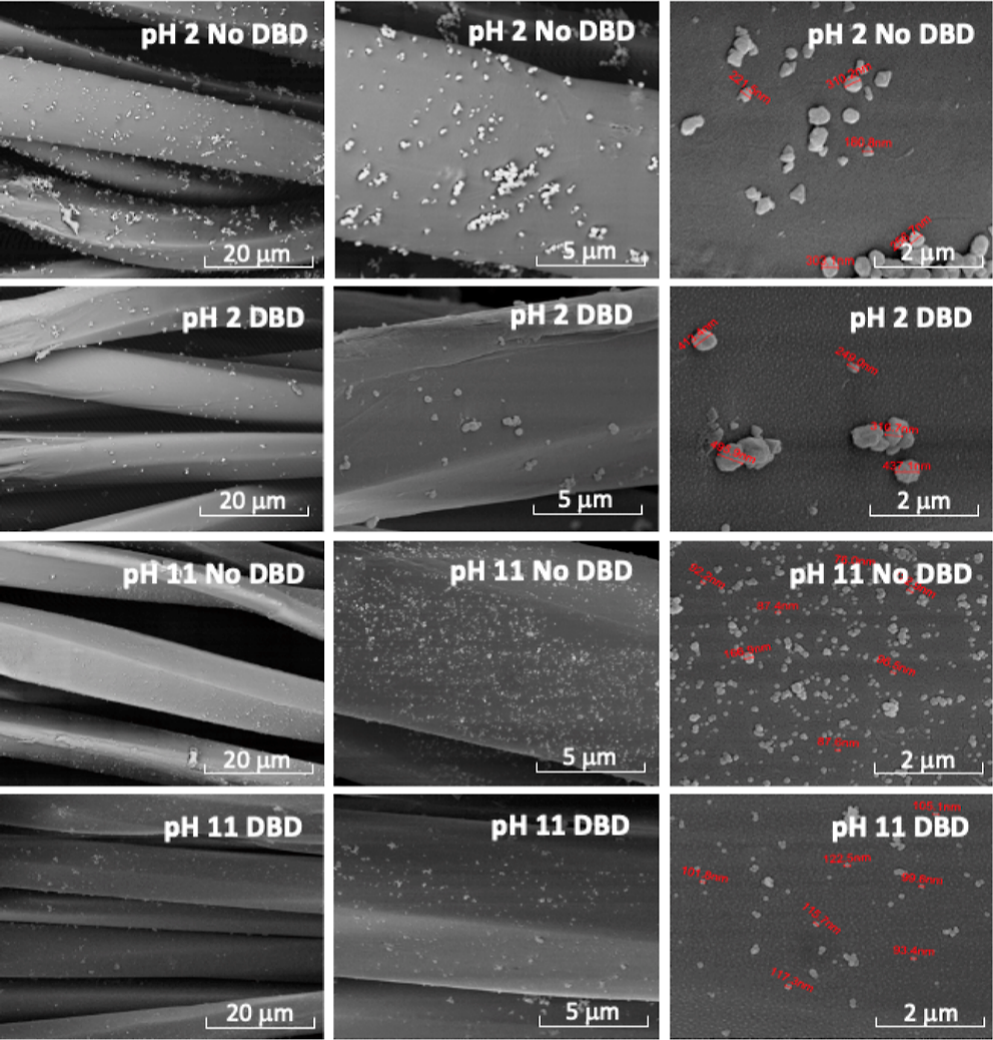
SEM images of PET samples functionalized in situ with the higher concentration of CuNPs in acidic (pH 2) or alkaline (pH 11) medium. DBD plasma-treated (DBD) and not treated (No DBD) PET samples were analyzed at magnifications of 5000×, 15 000×, and 50 000×.

The surface morphology of the PET fabric after the in situ synthesis of CuNPs was studied by SEM with different magnifications. Images were collected for samples prepared with a higher concentration of copper salt (Figure 2) and for the samples prepared with low-concentration solutions in the in situ synthesis. For the samples with a lower concentration of copper, images after 5W and 10W cycles were also taken (Figures S1 and S2 in the Supporting Information).

The SEM images of samples with the higher concentration of CuNPs showed well-distributed nanoparticles in all samples. However, some differences may be observed in the samples prepared using the acidic and alkaline syntheses, as well as in the samples prepared with and without DBD plasma treatment. In the acidic reaction, the SEM images of the sample without DBD pretreatment (pH 2-No DBD) have displayed a good dispersion with minor agglomerations of CuNPs onto the PET fabric’s surface. The size of CuNPs varied from 118.0 to 310.2 nm. Quasispherical and disk-like shapes were observed. In the sample with DBD plasma treatment (pH 2-DBD), the SEM images showed higher agglomeration, and the size increased to the range between 249 and 500 nm. In the alkaline reaction, the SEM images of the sample without DBD plasma treatment have displayed very well-dispersed CuNPs with negligible agglomeration, and the size was in the range of 76 to 157 nm. The pH 11-DBD sample exhibited good dispersion of CuNPs on the fabric’s surface with minor agglomerations, and the size ranges from 93.4 to 123.0 nm. The size of the CuNPs in the acidic medium was bigger than the size of CuNPs in the alkaline reaction. These findings followed the explanation given by Biçer and Şişman that the size of particles is inversely proportional to the pH value. The AA has a higher reduction tendency at higher pH values, explaining the abovementioned behavior.^37^ The reduced size of CuNPs in the alkaline reaction can also be due to the activity of NaOH as a reducing agent.^37^ Despite the fiber modification performed in this work, neither the DBD plasma treatment nor the alkaline reaction was found to considerably decrease the mechanical properties of the PET fabric (Figure S3 in the Supporting Information).

The samples prepared using a lower concentration of copper salt also presented well-dispersed CuNPs despite being less in number (Figure S1 in the Supporting Information). Even after 10W cycles, some CuNPs remain in the PET fabric (Figure S2 in the Supporting Information).

#### 2.2.2 Color Measurements

**Figure 3:**
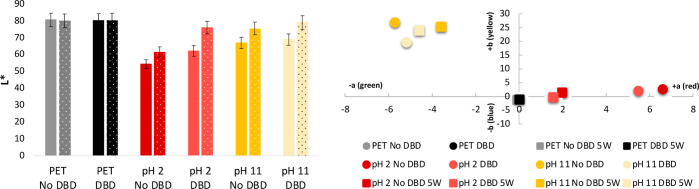
Color coordinates of the PET samples (L*—lightness values, a*—yellowness–blueness, and b*—redness–greenness) functionalized with in situ CuNPs in acidic or alkaline medium using the initial concentration of copper salt (10 mM) before and after 5W cycles (L*—dotted bars and a* and b*—5W); PET samples with (DBD) and without DBD plasma treatment (No DBD) were used as control.

The color of the PET samples was assessed in the *Commission Internationale de l’Elcairage* (CIE) L* (lightness), a* (redness–greenness), and b* (yellowness–blueness) space before and after 5W cycles using the initial concentration of the solution (10 mM of copper salt) and the 5TD solutions (2 mM of copper salt) (Figure 3). The colors of the samples were red when the CuNPs were prepared under acidic conditions or yellow when an alkaline medium was used. The plasma-treated samples presented similar color coordinates, but the lightness values were found to be higher, which can be due to the superior agglomeration of CuNPs when DBD plasma treatment was used. After 5W cycles, the lightness of the samples increased compared with the non-washed samples. When a minor concentration of copper salt was used in the reaction, the colors faded. In the samples prepared using the 5TD solution, it was still possible to have a red (pH 2) or yellow (pH 11) shade (Figure S4 in the Supporting Information). Samples using 10TD solutions were also prepared, but any visual differences could be detected in the samples using less concentration of copper salt or the corresponding washed samples.

Interestingly, the sample pH 2 DBD showed a lower lightness value, and a more positive a* emerged than that of the other 5TD samples (Figure S4 in the Supporting Information). After 5W cycles, the a* value increased more. These results suggested the superior concentration and diffusion of CuNPs onto the fabric using DBD plasma treatment. The higher a* value after washing can be justified by the migration of the CuNPs from the interior of the fabric to the surface, promoted by the washing process.

#### 2.2.3 Assessment of UV Protection

The UV-transmittance was measured to calculate the UV- protection of the PET samples according to the standard AATCC 183:2020. The UV protection factor (UPF) was calculated in samples with a higher concentration of CuNPs and the samples prepared using 5TD solutions (Table 1). The results showed that the UPF value was proportional to the concentration of CuNPs. Samples with a higher concentration of CuNPs presented superior values than 5TD samples and the control samples. Despite the control samples already display excellent UPF values, the CuNPs enhanced, even more, the light reflection and scattering, and consequently the UPF increased.^44^

**Table 1:** UV-Protection Results of the Samples Prepared at pH 2 and 11, and Control Samples with the Initial Concentration of Copper Salt (10 mM) and 5TD Solutions (2 mM) before and after 5W Cycles.

	prewashing			postwashing (5W)		
	UV-A	UV-B	UPF	UV-A	UV-B	UPF
PET No DBD	13.4	0.5	56.2	13.1	0.5	57.2
PET DBD	13.2	0.5	56.8	13.1	0.5	56.7
pH 2 No DBD	2.7	0.1	249.7	4.5	0.2	156.2
pH 2 DBD	6.2	0.3	110.7	9.5	0.4	79.1
pH 11 No DBD	0.6	0.1	655.5	2.8	0.1	222.6
pH 11 DBD	2.4	0.1	248.6	4.0	0.2	170.2
pH 2 No DBD-5TD	10.27	0.41	71	12.17	0.51	61
pH 2 DBD-5TD	9.28	0.39	77	10.33	0.46	69
pH 11 No DBD-5TD	11.39	0.45	64	12.86	0.53	57
pH 11 DBD-5TD	11.67	0.49	63	13.20	0.58	54

In the samples with the higher concentration of CuNPs, the UPF increased from 56.2 to 56.8 for untreated PET to 110.7 and 655.5 for samples with CuNPs. Samples containing DBD plasma treatment showed lower values than untreated samples, related to the superior agglomeration when DBD was applied. The UPF of samples prepared in acidic medium (pH 2) decreased from 249.7 to 110.7, and in the samples prepared in alkaline medium (pH 11) the UPF decreased from 655.5 to 248.6 when DBD plasma treatment was applied. Some works have shown the relation between the size of MNPs and the corresponding UV protection, where the smaller size of MNPs corresponds to superior UV protection.^45,46^ Overall, the sample pH 11-No DBD showed the greatest UPF value. After 5W cycles, the UPF decreased in all samples, but excellent result was maintained.

When a lower concentration of copper salt in the synthesis was used (5TD samples), the UPF considerably decreased compared with that of the samples with the higher CuNP concentration, revealing a UPF between 63 and 77. Here, the sample prepared with DBD plasma treatment in acidic pH presented superior UV protection (77) than the untreated samples (71) and also than the alkaline-prepared samples (64–63). After 5W cycles, the sample with DBD plasma treatment (pH 2-DBD) still had superior UV protection. Despite the bigger CuNP size in this sample, the superior concentration of CuNPs in samples with DBD plasma treatment and their migration to the surface with the washing could promote the superior UPF.

#### 2.2.4 TGA Analysis

**Figure 4:**
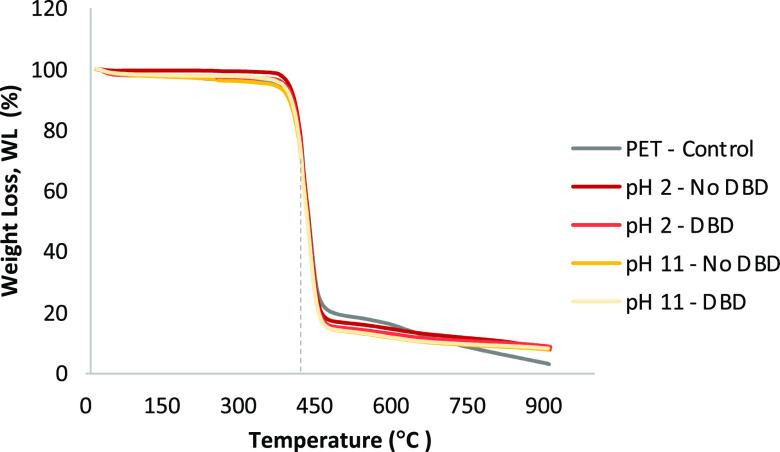
TGA thermograms of the PET with CuNPs prepared in acidic (pH 2) and alkaline (pH 12) reactions, and PET without CuNPs as a control sample (PET—control).

TGA analyses were performed to identify the phase transitions of the samples and identify the content of CuNPs in the bulk samples using the residue values (*R*) (Figure 4). The weight loss (WL) curves showed the percentage of loss as a function of temperature between 25 and 900 °C. The main WL emerged at 423 °C in all the samples, independent of the CuNP concentration. This thermal degradation was attributed to PET. The *R* were calculated by subtracting the initial weight of the samples at 25 °C from the final weight at 900 °C. Then, the residue variations were assessed by subtracting the residue percentage of the control sample (PET—control, 2.49%) from the percentage of each sample containing CuNPs. These calculations make it possible to observe a superior concentration of CuNPs on samples with DBD plasma treatment. The reaction in acidic media with DBD plasma treatment presented more CuNPs. The CuNP content increased from 4.94 to 5.97% using plasma. Also, in alkaline media, the DBD improved the adhesion of CuNPs with an increase from 4.90 to 5.39%. The TGA analysis allows obtaining the total content of CuNPs in each sample and not just in the surface, showing a superior diffusion of the CuNPs in the DBD plasma-treated samples. This difference is much more pronounced at acidic pH.

#### 2.2.5 XPS Analysis

**Figure 5:**
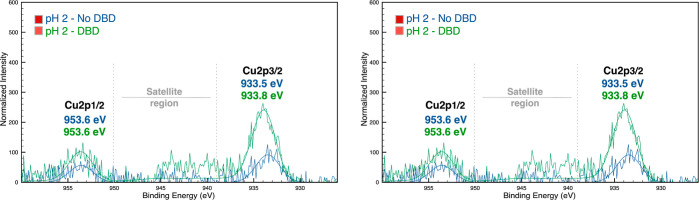
Cu 2p high-resolution spectra of the samples in acidic and alkaline pH in PET DBD plasma-treated and not treated samples.

**Figure 6:**
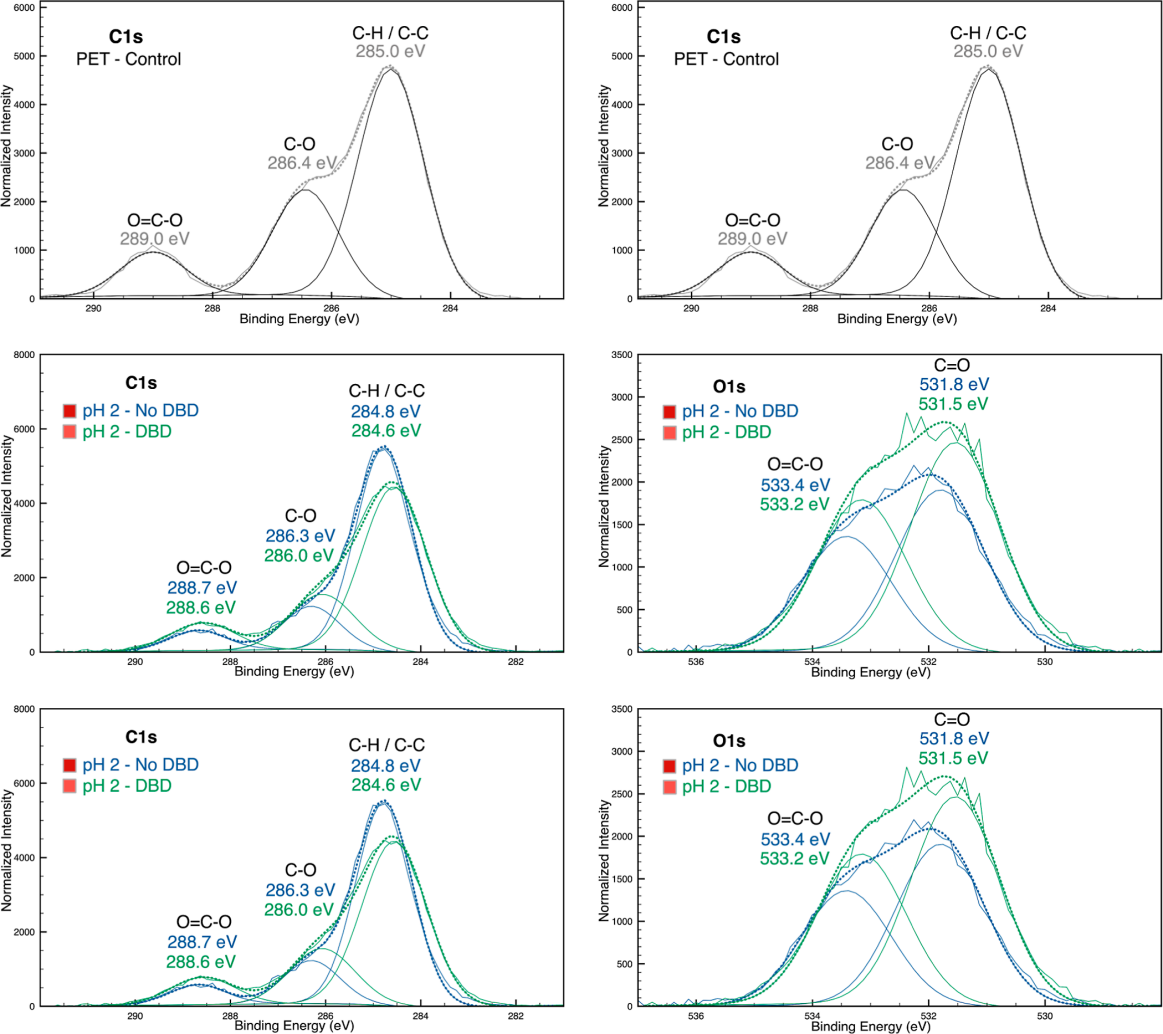
C 1s and O 1s high-resolution spectra of the control PET and the samples in acidic (pH 2) and alkaline pH (pH 11) in DBD plasma-treated and untreated PET.

The chemical composition and elemental bonding on the samples’ surface were demonstrated by XPS analysis. Samples prepared using the initial concentration of copper salt were analyzed. The survey spectra (Figure S5 in the Supporting Information) and high-resolution spectra of Cu 2p, C 1s, and O 1s were obtained and deconvoluted (Figures 5 and 6).

The Cu 2p high-resolution spectra confirmed the presence of copper on the surface of all functionalized samples (Figure 5). Two different peaks emerged in the high-resolution spectra of Cu 2p, which corresponded to Cu 2p_3/2_ and Cu 2p_1/2_ of the copper species.^38,47−49^ In acidic pH (pH 2-No DBD and pH 2-DBD samples), the peaks appeared from 933.5 to 933.8 and at 953.6 eV, and in the alkaline samples (pH 11-No DBD and pH 11-DBD samples), the peaks appeared from 932.7 to 933.5 and 952.4–953.1 eV. The Cu 2p_3/2_ peaks in the DBD plasma-treated samples (pH 2-DBD and pH 11-DBD) emerged at slightly higher binding energy than those of the corresponding untreated samples. This suggested additional oxidation in the samples’ surface when DBD plasma treatment was applied. Under acidic conditions, the lack of strong shake-up satellites between 940 and 945 eV, attributed to the Cu^2+^ species, suggests that the main oxidized specie is Cu_2_O. However, it is not excluded that few CuO species may be present on the surface of the PET fabric.^50^ Under alkaline conditions, the small satellite peak clearly shifted to higher binding energies, confirming the presence of only Cu_2_O on the fabric surface. The superior oxidation of CuNPs when DBD plasma treatment was applied was not a surprise. Several species containing atomic oxygen, nitrogen oxides, ozone, neutral and metastable molecules, and radicals are produced during the treatment. For these reasons, the surface energy of PET treated with DBD may increase, facilitating the functionalization.^51^ Despite the evidence, it was challenging to distinguish the Cu metal and copper oxide species using XPS only. Thus, the XRD analysis was performed in these samples to understand the obtained oxidation state of copper during the synthesis.

In addition, some information can be obtained regarding the concentration of CuNPs on the fabrics’ surface. The superior intensity of the peaks suggested a higher concentration of CuNPs on the surface of pH 2-DBD and pH 11-No DBD samples (Figure 5). The DBD plasma treatment under the acidic conditions promoted superior adhesion of the NPs onto the PET surface. On the contrary, the sample without DBD presented a superior concentration of CuNPs on the PET surface in the alkaline reaction.

The characteristic peaks attributed to the PET fabric before and after functionalization were observed in the C 1s and O 1s high-resolution spectra of the samples, and sensitive information was provided (Figure 6). In the C 1s spectra of the control PET sample, three peaks appeared at 285.0, 286.5, and 289.0 eV, corresponding to C–C/C–H, C–O, and O=C–O, respectively. In the O 1s spectra, the main peaks were deconvoluted into two components at 532.0 and 533.6 eV attributed to C=O and O=C–O bonds.^52^

As suggested above, DBD plasma treatment and the pH of the medium changed the available groups on the PET surface. In the samples prepared at pH 2, the C 1s high-resolution spectra showed an increased intensity of the C–O and O=C–O peaks when DBD plasma treatment was applied. Similarly, the O 1s spectra showed the same pattern. The O/C ratio was 0.18 in the pH 2-No DBD sample and 0.25 in the pH 2-DBD sample. However, in DBD plasma-treated samples prepared at alkaline pH, the same groups decreased in intensity, confirmed by the O/C ratio of 0.24 to pH 11-No DBD and 0.19 to pH 11-DBD samples. The alkaline reaction neutralized some species formed during DBD plasma treatment, and the oxygen component was superior in not treated samples. However, comparing the control sample and pH 11-No DBD sample, it was possible to observe a slight deviation of the peak at 532.0 to 531.4 eV, suggesting the emergence of oxygen containing groups with low binding energy, such as hydroxyl groups.

#### 2.2.6 XRD Analysis

**Figure 7:**
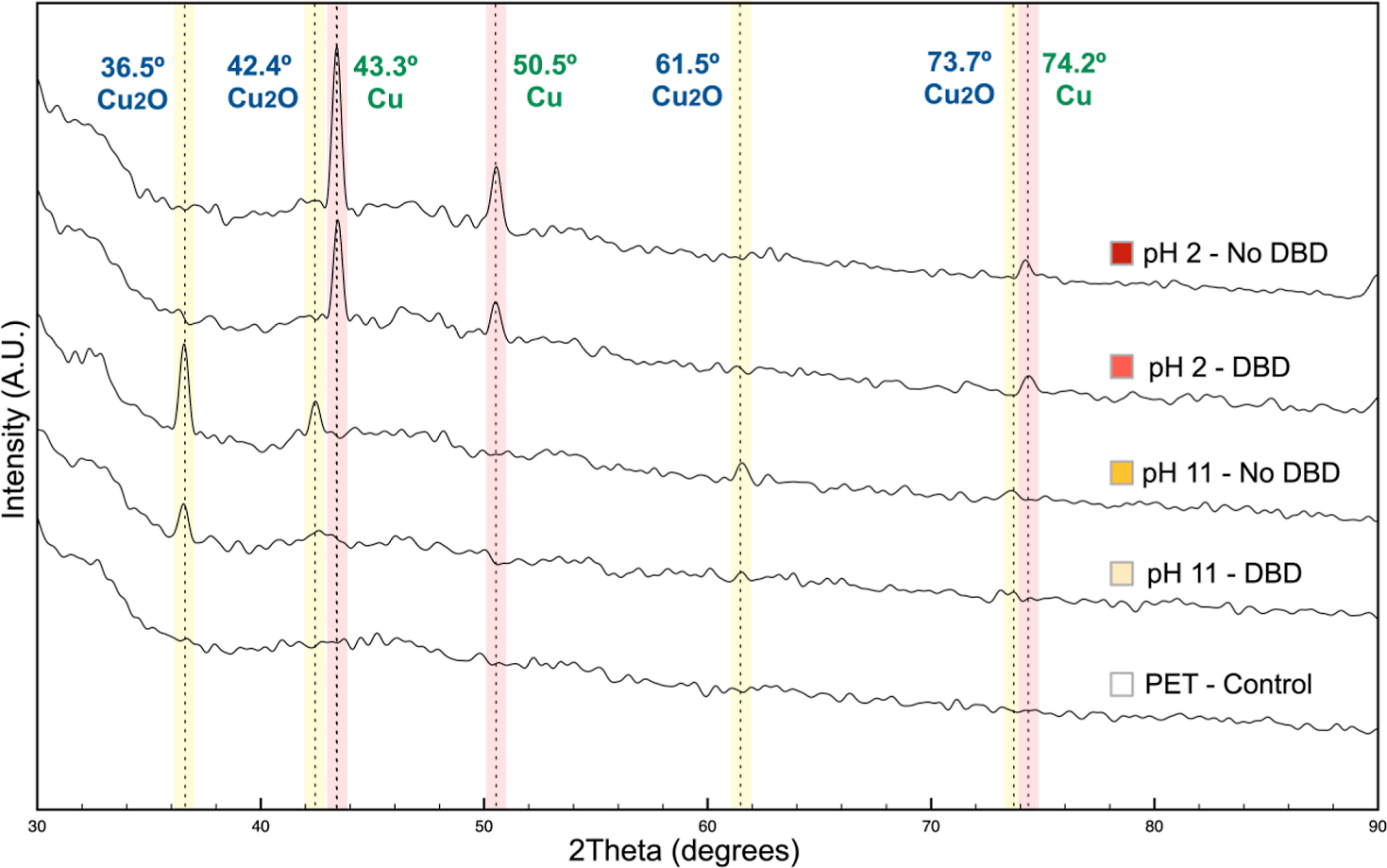
XRD spectra of the PET fabric (control) and samples with CuNPs prepared in acidic and alkaline reactions.

XRD analysis was performed to confirm the crystal phase change of synthesized CuNPs in acidic and alkaline reactions (Figure 7). The XRD patterns of samples prepared in the acidic medium corresponded to metallic copper with peaks at 43.4, 50.5, and 74.2°, according to existing references (PDF 03-065-9026 and PDF 00-001-1242), related to the crystal facets of (111), (200), and (220), respectively. Moreover, when the alkaline medium was used for CuNP synthesis, the XRD pattern showed the presence of copper(I) oxide with peaks at 36.5, 42.4, 61.5, and 73.7°, according to the references (PDF 01-071-4310 and PDF 01-073-6237), related to the crystal facets of (111), (200), (220), and (311), respectively. DBD plasma-treated samples showed the same XRD patterns as the not treated samples. The Cu(II) species suggested by XPS analysis were not detected in XRD analysis, which could be justified by the depth of the analysis. The average depth of XPS analysis is 10 nm, whereas for XRD analysis it is up to 10 mm. Thus, the superior oxidation on the PET surface was not provided by the synthesis of CuNPs but by DBD plasma treatment or by air surface oxidation, which acts only on the fabric surface.

#### 2.2.7 Antibacterial Analysis

American Society for Testing and Materials (ASTM) E2149 standard was used to assess the antibacterial performance of the PET samples functionalized with CuNPs at different concentrations against *S. aureus* and *E. coli* bacteria. Samples using the initial concentration of copper salt (CuSO_4_·5H_2_O 10 mM solution), 5 TD solutions (CuSO_4_·5H_2_O 2 mM solution), and 10 TD solutions (CuSO_4_·5H_2_O 1 mM solution) were prepared to find the optimal concentration of copper to obtain an effective antibacterial effect. The samples were tested before and after washing cycles (5W or 10W) to quantify indirectly the release of CuNPs.

**Figure 8:**
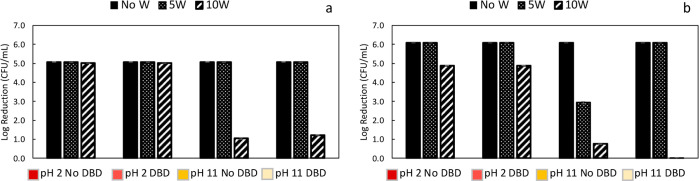
Antibacterial activity of PET samples against *S. aureus* (a) and *E. coli* (b) before and after 5W and 10W cycles and samples prepared with 5TD solutions (2.0 mM of copper salt).

Using the initial concentration of copper salt, all samples exhibited an excellent antibacterial effect against both bacteria (log reduction of 5) (Figure S6 in the Supporting Information). Similarly, the samples using the 5TD solutions also showed remarkable antibacterial efficacy in the acidic reaction, even after 10W cycles. In the alkaline reaction, the antibacterial efficacy remains until 5W cycles. In this case, DBD plasma treatment displayed a significantly different effect against *E. coli* (Figure 8). The sample without DBD plasma treatment showed a log reduction of 2.9 ± 0.1, while the corresponding DBD plasma-treated sample showed a log reduction of 6.1, underscoring the ability of plasma to enhance the adhesion of CuNPs when washings were performed. Different results were obtained when 10 washings were performed. The samples prepared in acidic medium maintained their bactericidal activity against *S. aureus* and *E. coli*. However, under alkaline conditions, the antibacterial effect considerably decreased (log reduction from 1.1 ± 0.1 to 1.2 ± 0.1 against *S. aureus*). No antibacterial effect was observed against *E. coli* (Figure 8).

Then, in the samples prepared using 10TD solutions (1.0 mM of copper salt), the antibacterial efficacy of most samples considerably decreased. Considering the standard deviation of the results in acidic medium and against *S. aureus*, no striking differences were obtained in samples with and without DBD. In the alkaline pH, before washings, the log reduction of the untreated sample was 2.2 ± 0.2, and with DBD plasma treatment it was 1.3 ± 0.1. After 5W cycles, the untreated sample showed a log reduction of 0.6 ± 0.2, while the DBD plasma-treated sample showed a log reduction of 2.9 ± 0.9 (Figure S7 in the Supporting Information). Against *E. coli*, the 10TD concentration was found to be ineffective.

Generally, the samples prepared in the acidic medium were found to be more effective against *S. aureus* and *E. coli* than the samples prepared in the alkaline reaction. Moreover, the DBD plasma treatment showed some influence to retain the antibacterial effect, showing a superior impact on the alkaline samples after 5W cycles.

Various mechanisms can explain the antibacterial activity of CuNPs: (i) nanoparticles can release Cu^2+^ ions, and they can be attached to the negatively charged bacterial cell wall that leads to the death of the cell; (ii) the large surface area of CuNPs facilitates the interaction between CuNPs and the bacterial membrane; and (iii) it is believed that CuNPs will act as an activating point for the destruction of the cell wall, generation of ROS, oxidation of proteins, peroxidation of lipids, and genome degradation and also prevent the replication of DNA in microorganisms.^42,53−56^

## 3 Conclusions

This study reported a facile and safe method to synthesize in situ CuNPs onto the PET fabric under alkaline and acidic reaction conditions. Performing the synthesis in acidic pH, a red color was obtained in the fabrics, and metallic copper was detected. When the synthesis was performed in alkaline pH, smaller and yellow CuNPs were obtained, but the copper’s oxidation state increased to Cu(I). A detailed investigation was carried out on the effects of DBD plasma treatment on the enhancement of the antibacterial performance of the samples, and good results were obtained in the alkaline synthesis of CuNPs. Using DBD plasma treatment, it was possible to reduce the CuNP concentration in the PET fabric maintaining an effective antimicrobial activity. The functionalized PET fabrics can potentially find several medical textile applications as UV-protective or antimicrobial textiles and sensors.

## 4 Experimental Section

### 4.1 Materials

Copper sulfate (CuSO_4_·5H_2_O), AA, and sodium hydroxide (NaOH) were purchased from Merck. SHP was obtained from Sigma-Aldrich. Commercial PET fabric (weight per unit area of 100 g·m^–2^ and warp/weft density of 29/31) was purchased from T-GRAMA (Braga, Portugal). To minimize the contaminations, the fabric was prewashed with a non-ionic detergent solution (1 g·L^–1^) at 60 °C for 60 min with a liquor ratio of 1:100, rinsed with distilled water, and dried at 40 °C.

### 4.2 DBD Plasma Treatment

A semi-industrial prototype machine (Softa GmbH/University of Minho) was used to conduct the DBD plasma treatment at room temperature and atmospheric pressure in air. It is a system of metal electrodes coated with ceramic and counter electrodes coated with silicon of 50 cm effective width, the gap distance fixed at 3 mm, and producing the discharge at a high voltage of 10 kV and a low frequency of 40 kHz. The discharge power supplied by the electrodes and the speed may change, with a maximum discharge of 1.5 kW and a speed of 60 m min^–1^. The machine was operated at optimized parameters, 1.0 kW of power and a velocity of 4.0 m min^–1^, for five passages in both sides of PET samples, corresponding to a dosage of 2.5 kW·min·m^–2^.^57^

### 4.3 In Situ Synthesis of CuNPs under Acidic and Alkaline Conditions

The in situ synthesis of CuNPs was carried out using three different concentrations from the starting solutions (Table S1 in the Supporting Information). The samples with a higher concentration of copper salt in acidic medium (pH 2) were prepared according to the following method: (i) distilled water (100 mL) was added into a round bottom flask and heated until 65 °C; (ii) CuSO_4_·5H_2_O (10 mM, 40 mL), SHP (28 mM, 30 mL), and AA (60 mM, 30 mL) were quickly added in this order into the round bottom flask; (iii) after adding these solutions, a light red color was noticed, which indicates the starting of the reduction reactions; (iv) the PET sample (5 × 5 cm) was immediately added into the flask, and the temperature was adjusted to 85 °C; and (v) the solution was left at 85 °C for 1 h, and the PET sample was collected, rinsed with distilled water, and dried at 40 °C. This procedure was performed using PET samples with and without DBD plasma treatment. The same procedure was followed starting from a solution of NaOH (44 mM, 100 mL) for the alkaline reaction (pH 11). The PET fabric was added immediately after the emergence of a yellow-brownish color. Also, samples with and without DBD plasma treatment were used. Samples without CuNPs and treated or not treated with DBD plasma treatment were used as control. Moreover, PET samples were prepared from 5TD and 10TD solutions of all reagents (Table S1 in the Supporting Information).

### 4.4 Washing Fastness

A laboratory-dyeing machine (Ahiba, Datacolor, Lawrenceville, New Jersey, USA) was used to assess the resistance to washing. The assessment was done after 5 and 10W cycles, and the apparatus was used at 75 °C and 40 rpm for 15 min with 0.1 g·L^–1^ of non-ionic surfactant in a liquor bath ratio of 1:30. After washing, the samples were rinsed five times at 30 °C for 15 min with distilled water to remove the remaining detergent.

### 4.5 Scanning Electron Microscopy

An ultrahigh resolution field-emission gun scanning electron microscope (NOVA 200 Nano SEM, FEI Company) was used to conduct morphological analyses of fabrics. Acceleration voltages of 5 and 15 kV were used to obtain secondary electron images and backscattering electron images, respectively. Samples were covered with a film of Au–Pd (80–20 weight %) in a high-resolution sputter coater, 208HR Cressington Company, coupled to an MTM-20 Cressington high-resolution thickness controller.

### 4.6 Measurement of Color

The color strength of the PET samples before and after the washing was studied using a UV-2600, UV–vis spectrophotometer manufactured by SHIMADZU, using a D65 illuminant in the range from 400 to 700 nm. The coloring effects in CIE L* (lightness), a* (yellowness–blueness), and b* (redness–greenness) space were calculated using RGB values. The transmittance data was used to calculate the UV radiation blocking for developed PET samples using the AATCC test method 183-2020.

### 4.7 Thermogravimetric Analyses

An STA 7200 Hitachi (Tokyo, Japan) was used to carry out the TGA analysis. TGA plots were obtained within the range of 25–900 °C under a nitrogen atmosphere (200 mL·min^–1^) at 10 °C·min^–1^. The samples were left at room temperature (25 °C) and were placed in an alumina pan. Data were plotted as WL percentage versus temperature, and the mass of dried residues was calculated. The maximum peaks of the thermal transformation events were identified by performing the derivative thermogravimetric analysis.

### 4.8 X-ray Photoelectron Spectroscopy

Kratos AXIS Ultra HSA (Manchester, UK) and VISION software were used to perform XPS analyses, and CASAXPS was used for data analysis. The analysis was carried out with a monochromatic Al Kα X-ray source (1486.7 eV), operating at 15 kV (150 W), in FAT mode (fixed analyzer transmission), with the pass energy of 40 eV for spectra of ROI and 80 eV for the survey spectrum. Pressure lower than 10^–6^ Pa was used to perform the data acquisition and it was used a charge neutralization system. Spectra have been charge-corrected with respect to the adventitious C 1s spectral component (C–C, C–H) with a binding energy of 285 eV. An analysis area of ∼1 mm^2^ was used to collect high-resolution spectra. The peaks were constrained to have equal full width at half-maximum to the prominent peak. This process has an associated error of ±0.1 eV. CASAXPS software (version 2.3.15) was used to analyze the spectra for elemental composition. Deconvolution into subpeaks was performed by least-squares peak analysis software, XPSPEAK version 4.1, using the Gaussian/Lorentzian sum function and Shirley-type background subtraction. In the peak-fitting procedure, no tailing function was considered. The components of the various spectra were mainly modeled as symmetrical Gaussian peaks unless a certain degree of Lorentzian shape was necessary for the best fit. The best mixture of Gaussian/Lorentzian components was defined based on the instrument and resolution (pass energy) settings used and the natural linewidth of the specific core hole.

### 4.9 X-ray Photoelectron Diffraction (XRD)

The PET samples containing the superior concentration of CuNPs were analyzed by XRD using a Bruker AXS D8 Discover diffractometer with a Cu Kα source (λ = 1.54060 Å). The analysis was carried out in the geometry θ/2θ at room temperature between 8 and 90°, with a step size of 0.04° and 5 s per step.

### 4.10 Antibacterial Properties

A positive Gram-bacterium *S. aureus* American Type Culture Collection (ATCC) 6538 and a Gram-negative bacterium *E. coli* ATCC 25922 were used to perform the antibacterial analyses. The antibacterial assays were performed following the ASTM-E2149-01 standard. Briefly, a preinoculum of each bacterium was prepared in tryptic soy broth and incubated for 12 h at 37 °C and 120 rpm. Afterward, each bacterium was centrifuged at 3900*g*, 18 °C for 10 min. The supernatant was discarded, and each bacterium was washed in sterile phosphate buffer saline (PBS) (pH 7.4) and subjected to another centrifugation cycle. Subsequently, each bacterium concentration was adjusted to approximately 1 × 10^7^ colony-forming units per milliliter (CFU mL^–1^) in PBS. 2 cm^2^ fabric was aseptically immersed in PBS containing the bacterium and incubated for 5 h at 37 °C and 120 rpm. Then, each bacterium from each sample was collected, subjected to serial dilutions, and incubated in tryptic soy agar for approximately 14 h at 37 °C. Afterward, the colonies were counted, and the CFU mL^–1^ was estimated.

## Supporting Information Available

Details of the conditions used for the in situ synthesis of CuNPs; additional SEM images and color coordinates of the samples prepared with lower concentrations of copper and washings; breaking force test of the fabrics; survey XPS spectra; and additional antibacterial tests (PDF)
