# Correction to “In Situ Synthesis of Copper Nanoparticles on Dielectric Barrier Discharge Plasma-Treated Polyester Fabrics at Different Reaction pHs”

**DOI:** 10.1021/acsapm.3c02545

**Published:** 2023-11-14

**Authors:** Behnaz Mehravani, Ana Isabel Ribeiro, Uros Cvelbar, Jorge Padrão, Andrea Zille

In our original paper, there are two errors:

(1) Figure 5: The second image on the right is a repetition of the first one on the left at pH 2. The correct figure is below.
(2) Figure 6: The image on the right of the first line is a repetition of the first one on the left. The third line is a repetition of the second line. The correct figure is below.

The corrections to the figures do not affect in any way the conclusion, discussion, or data interpretation of the work since the misplacement of the images was due to an editorial error. No other errors are present in the published paper.

**Figure 5:**
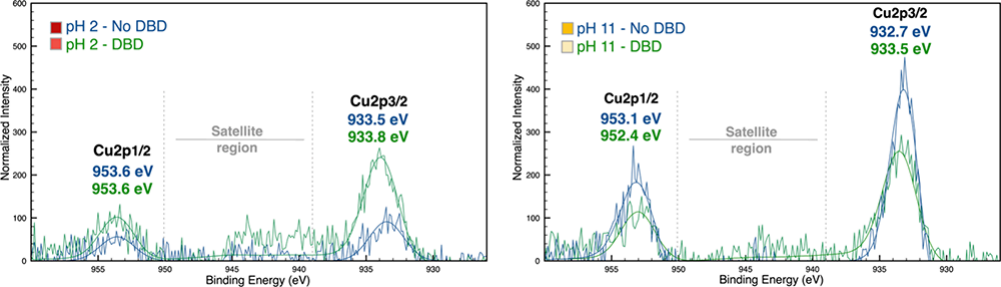
Cu 2p high-resolution spectra of the samples in acidic and alkaline pH in PET DBD plasma-treated and not treated samples.

**Figure 6:**
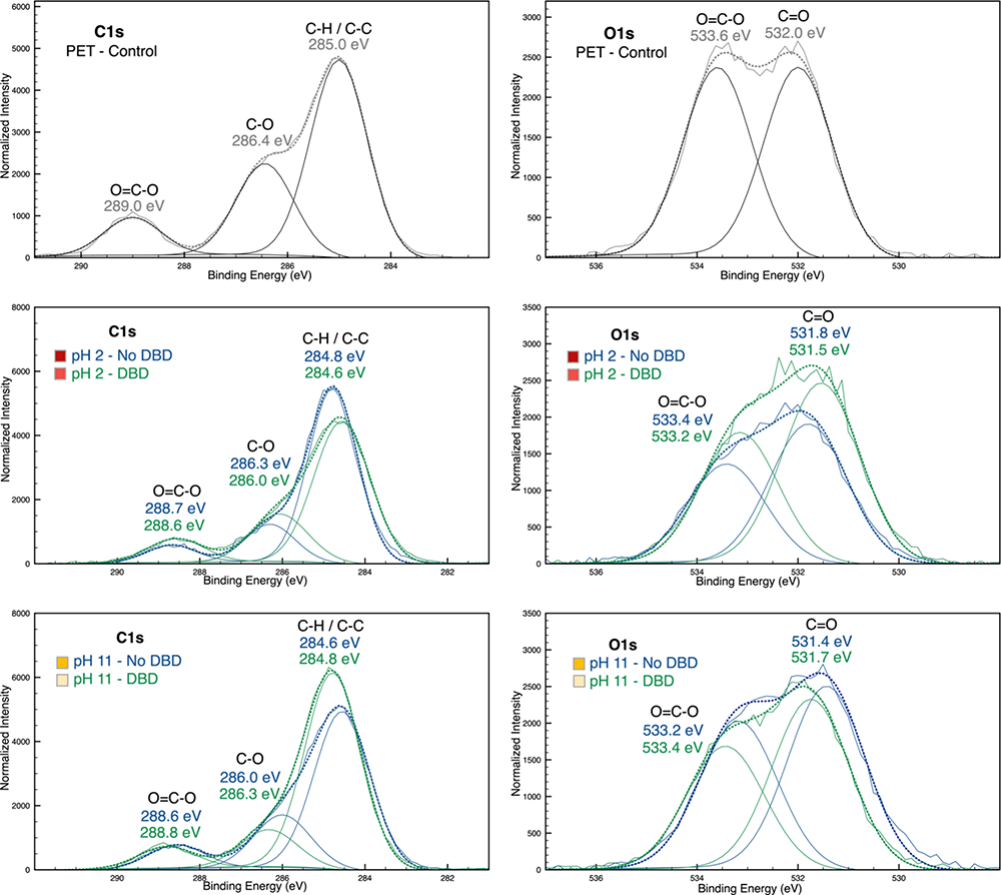
C 1s and O 1s high-resolution spectra of the control PET and the samples in acidic (pH 2) and alkaline pH (pH 11) in DBD plasma-treated and untreated PET.

