# Supplementary material for: In Situ Synthesis of Copper Nanoparticles on Dielectric Barrier Discharge Plasma-Treated Polyester Fabrics at Different Reaction pHs
Source: ACS Appl Polym Mater. 2022 Apr 15;4(5):3908–18. doi: 10.1021/acsapm.2c00375 (PMC9778008; doi:10.1021/acsapm.2c00375)
Supplement: Supplementary file 1 — ap2c00375_si_001.pdf [file ap2c00375_si_001.pdf]

SUPPORTING INFORMATION

In Situ Synthesis of Copper Nanoparticles on Dielectric Barrier Discharge Plasma Treated Polyester Fabric Under Different Reaction pH

Behnaz Mehravani[†,+], *Ana Isabel Ribeiro*[†,+], *Uros Cvelbar*[‡,§], *Jorge Padrão*[†] *and Andrea Zille*[*†]

[†]2C2T - Centre for Textile Science and Technology, Department of Textile Engineering, University of Minho, Campus de Azurém, 4800-058 Guimarães, Portugal

[‡]Department of Gaseous Electronics (F6), Jožef Stefan Institute, Ljubljana SI-1000, Slovenia

[§]Faculty of Mathematics and Physics, University of Ljubljana, Ljubljana SI-1000, Slovenia

[+] These authors contributed equally

[*]Corresponding author: E-mail: azille@2c2t.uminho.pt (Andrea Zille)

Table S1. Different conditions used for CuNPs synthesis: i) using an initial concentration of copper salt and corresponding reducing agents, or ii) 5 times diluted solutions (5TD) or iii) 10 times diluted solutions (10TD).

Code	$CuSO_4.5H_2O$		SHP		AA		dH_2O	NaOH	
	Concentration (mM)	Volume (mL)	Concentration (mM)	Volume (mL)	Concentration (mM)	Volume (mL)	Volume (mL)	Concentration (mM)	Volume (mL)
pH 2	10.0	40	28.0	30	60.0	30	100	-	100
pH 2 5TD	2.0	40	5.6	30	12.0	30	100	-	100
pH 2 10TD	1.0	40	2.8	30	6.0	30	100	-	100
pH 11	10.0	40	28.0	30	60.0	30	-	44.0	100
pH 11 5TD	2.0	40	5.6	30	12.0	30	-	8.8	100
pH 11 10TD	1.0	40	2.8	30	6.0	30	-	4.4	100

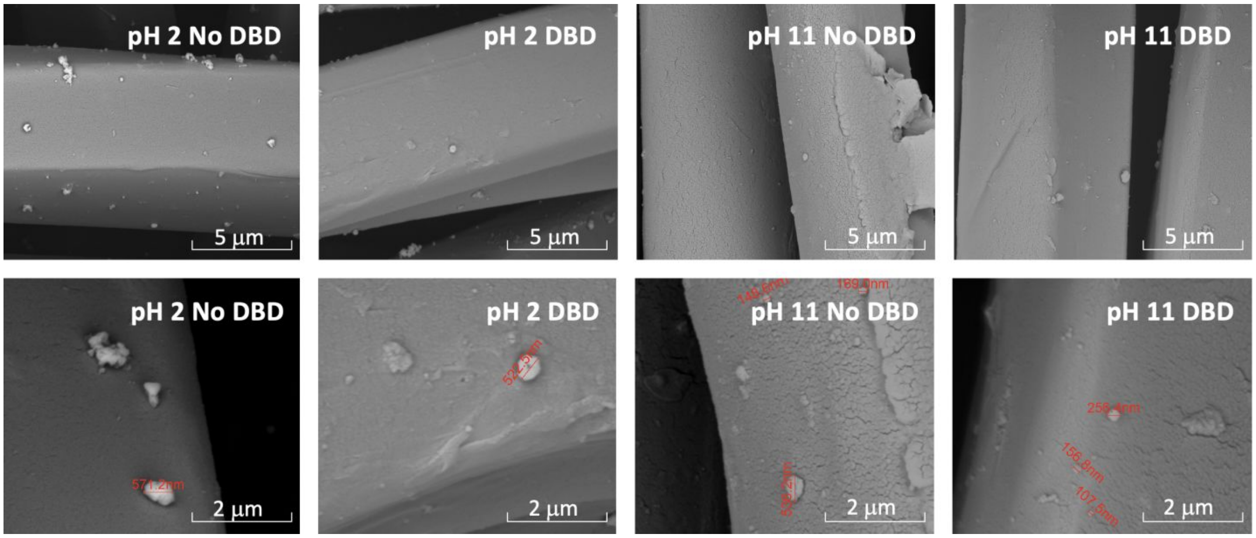

Figure S1. SEM images of PET samples functionalized *in situ* with a low concentration of CuNPs (5 times diluted solution - 5TD) in acidic (pH 2) or alkaline (pH 11) medium at a magnification of 15 000x and 50 000x.

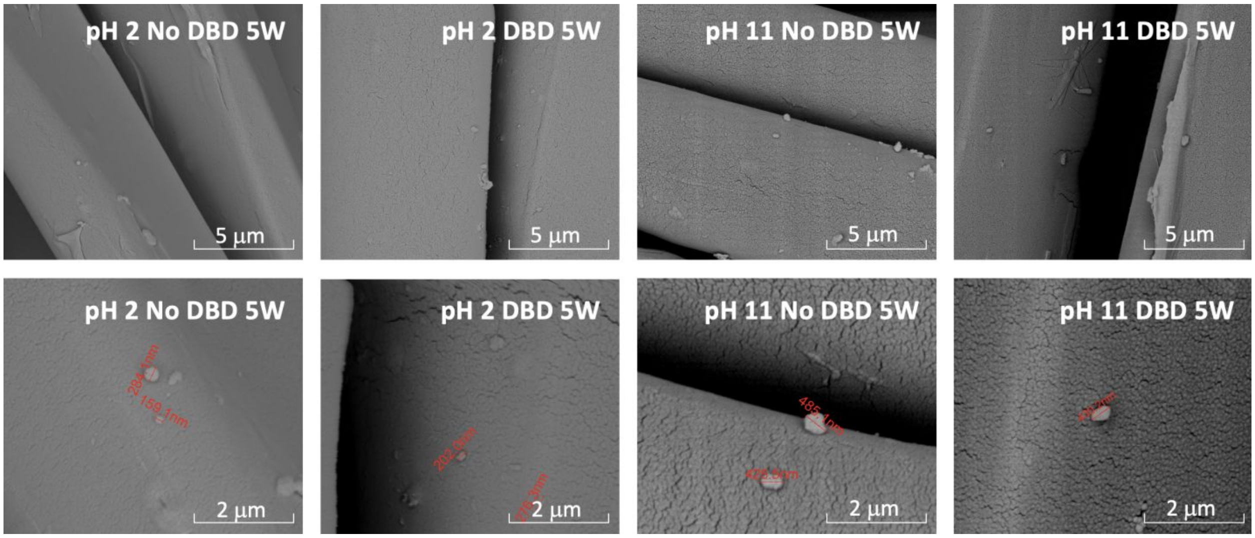

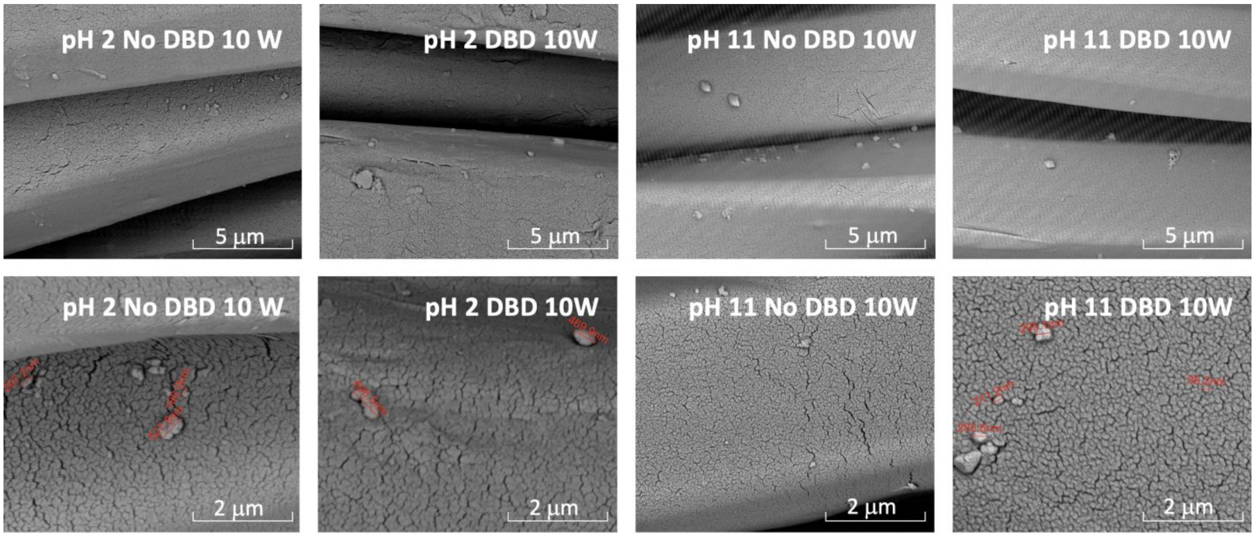

Figure S2. SEM images of PET samples functionalized *in situ* with a low concentration of CuNPs (5 times diluted solution - 5TD) in acidic (pH 2) or alkaline (pH 11) medium after 5 (5W) and 10 (10W) washing cycles at a magnification of 15 000x and 50 000x.

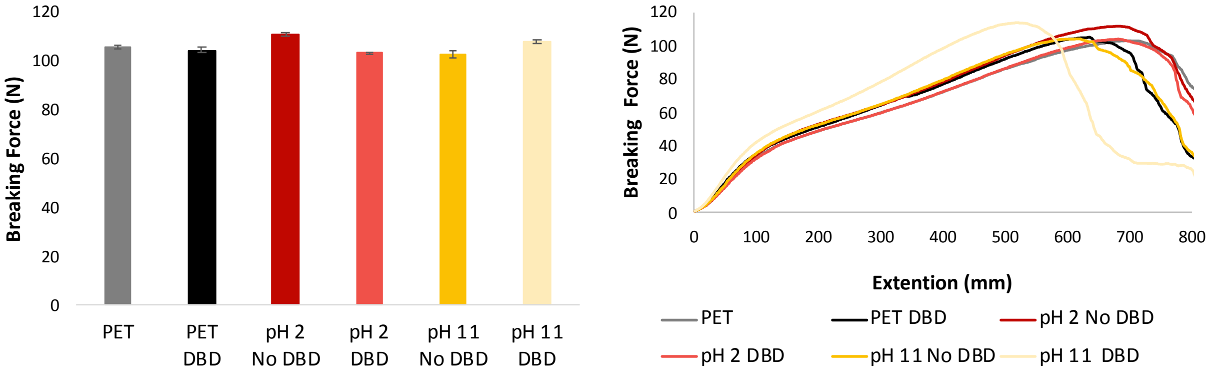

Figure S3. Breaking force of the samples according to ASTM D5035-11(2019) - standard test method for breaking force and elongation of textile fabrics (strip method).

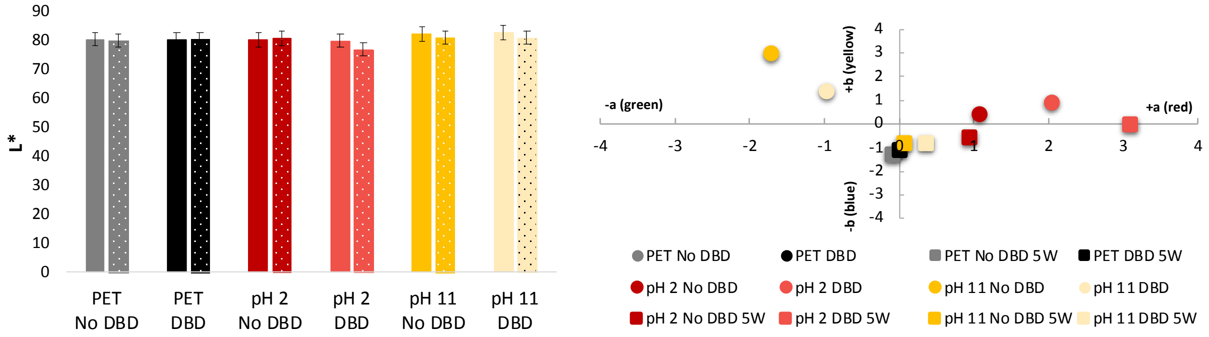

Figure S4. Color coordinates of the PET samples (L* - lightness values, a* - yellowness-blueness and b* - redness-greenness) functionalized with *in situ* CuNPs in acidic or alkaline reactions using 5 times diluted solutions (5TD, 2 mM) before and after 5 washing cycles (L* - doted bars and a* and b* - 5W); PET samples with (DBD) and without DBD plasma treatment (No DBD) were used as control.

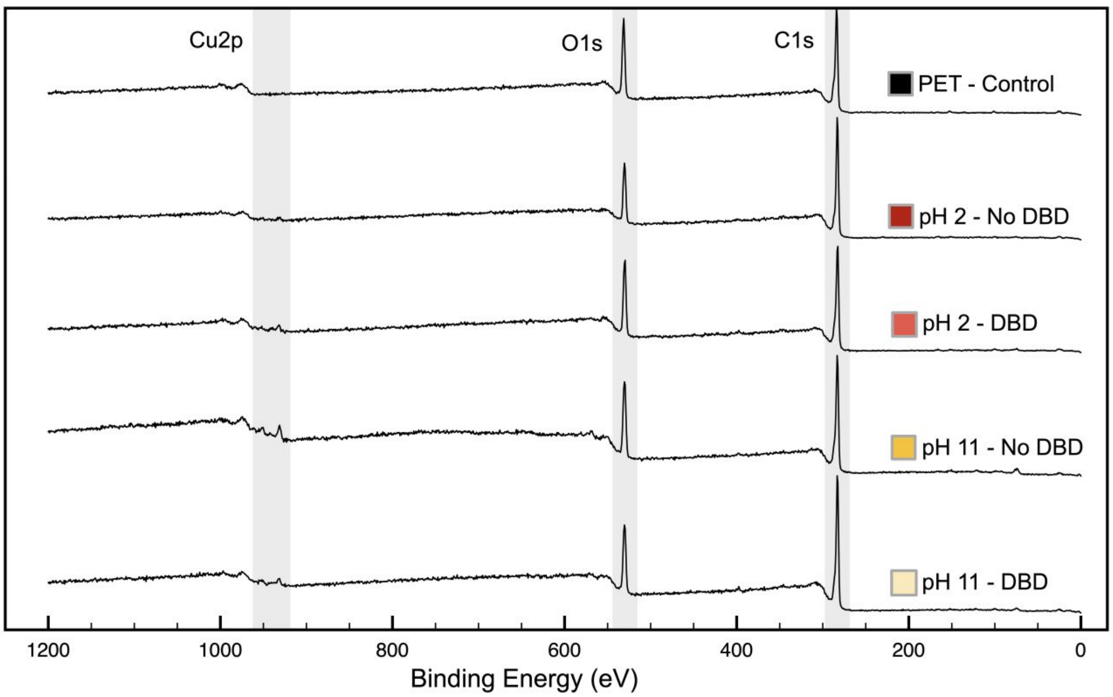

Figure S5. Survey spectra of control PET and PET samples with *in situ* synthesized CuNPs in acidic (pH 2) and alkaline (pH 11) reaction.

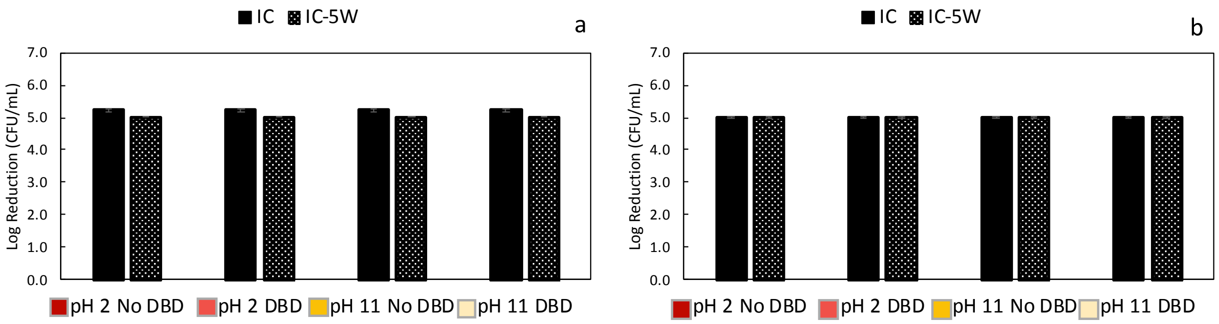

Figure S6. Antibacterial action of PET samples against *S. aureus* (a) and *E. coli* (b), before and after 5 washing cycles using samples prepared with the initial concentration of copper salt in CuNPs synthesis (IC, 10.0 mM of copper salt).

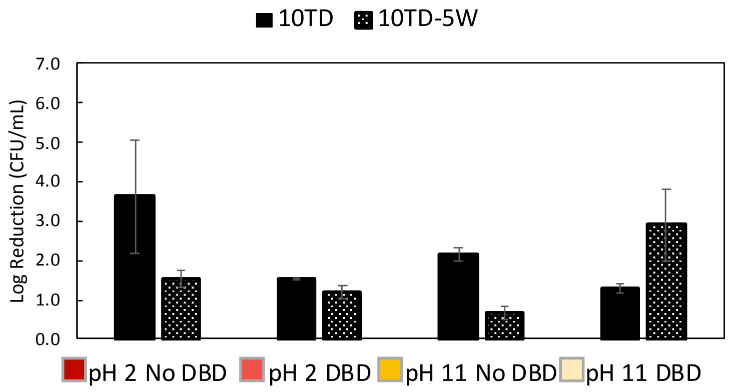

Figure S7. Antibacterial action of PET samples against *S. aureus*, before and after 5 washing cycles (5W) using samples prepared with the 10 times diluted solutions (10TD, 1.0 mM of copper salt).
